# Platelets and lymphocytes drive progressive penumbral tissue loss during middle cerebral artery occlusion in mice

**DOI:** 10.1186/s12974-021-02095-1

**Published:** 2021-02-18

**Authors:** Michael K. Schuhmann, Michael Bieber, Maximilian Franke, Alexander M. Kollikowski, David Stegner, Katrin G. Heinze, Bernhard Nieswandt, Mirko Pham, Guido Stoll

**Affiliations:** 1 Department of Neurology, University Hospital Würzburg, Würzburg, Germany; 2 Department of Neuroradiology, University Hospital Würzburg, Würzburg, Germany; 3 Rudolf Virchow Center for Integrative and Translational Bioimaging, University of Würzburg, Würzburg, Germany; 4 Institute of Experimental Biomedicine, University Hospital Würzburg, Würzburg, Germany

**Keywords:** Ischemic penumbra, Glycoprotein receptor Ib, T-cells, Ischemic stroke, Thrombo-inflammation, middle cerebral artery occlusion

## Abstract

**Background:**

In acute ischemic stroke, cessation of blood flow causes immediate tissue necrosis within the center of the ischemic brain region accompanied by functional failure in the surrounding brain tissue designated the penumbra. The penumbra can be salvaged by timely thrombolysis/thrombectomy, the only available acute stroke treatment to date, but is progressively destroyed by the expansion of infarction. The underlying mechanisms of progressive infarction are not fully understood.

**Methods:**

To address mechanisms, mice underwent filament occlusion of the middle cerebral artery (MCAO) for up to 4 h. Infarct development was compared between mice treated with antigen-binding fragments (Fab) against the platelet surface molecules GPIb (p0p/B Fab) or rat immunoglobulin G (IgG) Fab as control treatment. Moreover, *Rag1*^*−/−*^ mice lacking T-cells underwent the same procedures. Infarct volumes as well as the local inflammatory response were determined during vessel occlusion.

**Results:**

We show that blocking of the platelet adhesion receptor, glycoprotein (GP) Ibα in mice, delays cerebral infarct progression already during occlusion and thus before recanalization/reperfusion. This therapeutic effect was accompanied by decreased T-cell infiltration, particularly at the infarct border zone, which during occlusion is supplied by collateral blood flow. Accordingly, mice lacking T-cells were likewise protected from infarct progression under occlusion.

**Conclusions:**

Progressive brain infarction can be delayed by blocking detrimental lymphocyte/platelet responses already during occlusion paving the way for ultra-early treatment strategies in hyper-acute stroke before recanalization.

**Supplementary Information:**

The online version contains supplementary material available at 10.1186/s12974-021-02095-1.

## Introduction

In acute ischemic stroke, the therapeutic success of recanalization by thrombolysis and/or mechanical thrombectomy (MTE) largely depends on the extent of structural brain damage before the intervention [1] and on ischemia/reperfusion (I/R) injury thereafter [2]. Numerous investigations addressed pathomechanisms underlying I/R injury in ischemic organs in general [3–5]. However, the fact that tissue damage rapidly progresses from the core region into the penumbra under the condition of vessel occlusion, and thus, before recanalization and reperfusion, has been largely neglected [6–8].

The survival of the ischemic penumbra, defined as dysfunctional, but still viable brain tissue under occlusion of a major cerebral artery, critically depends on residual collateral blood flow. At present, it is unclear whether collateral blood flow exhausts primarily [9–12] or secondarily as a consequence of increased microvascular resistance which would physically impair perfusion pressure [13]. Alternatively inflammatory processes underlie progressive neuronal death. It has been demonstrated in experimental stroke models that the microcirculation is immediately affected during cerebral ischemia [14]. Accordingly, studies by the groups of Hallenbeck and Del Zoppo showed that early in cerebral ischemia platelets accumulate in the microvasculature and form aggregates with leukocytes, but do not completely obstruct the microvessels [15, 16]. Similarly, in hyperacute human stroke, leukocytes accumulate within the secluded vessel territory distal to MCA occlusion [17]. At present, it is unclear, whether these platelet/leukocyte accumulations functionally contribute to primary infarct growth. To address this issue, we took a particular experimental approach by step-wise increasing time intervals of occlusion duration to determine primary infarct growth under occlusion. Based on findings in pathophysiological different ischemia/reperfusion paradigms of cerebral ischemia [2, 18, 19], we addressed the role of platelet glycoprotein Ib (GPIb) in conjunct with T-cells during MCA occlusion. To the best of our knowledge, this is the first proof of principle study in young male mice demonstrating that progressive infarction under occlusive condition is amenable to anti-platelet treatment. Moreover, we provide evidence that infarct progression under occlusion and I/R injury are not fundamentally different processes but are at least partly due to a continuum of detrimental thrombo-inflammatory events commencing immediately upon ischemia, but inevitably persisting into the reperfusion phase.

## Methods

### Animals

We randomized male C57Bl/6 N/J and *Rag1*^*−/−*^ mice (6–8 weeks old) and subjected them to a permanent or transient middle cerebral artery occlusion (MCAO) [20]. Animal studies were approved by the district government of lower Franconia and were conducted in accordance with the US National Institutes of Health Guide for the Care and Use of Laboratory Animals. The experiments were designed, performed, and reported according to the Animal Research: Reporting of In Vivo Experiments guidelines [21]. All C57Bl/6 N/J and *Rag1*^*−/−*^ mice were purchased from Charles River Laboratories (Sulzfeld, Germany).

### Ischemia model

Focal cerebral ischemia was induced by a 2, 3, or 4 h MCAO or a 2 h MCAO with a 6 h reperfusion phase [20]. Mice for all animal experiments were randomized and coded by an independent researcher who was not involved in data analysis, so experiments were carried out blindly. Investigators involved in the surgery and evaluation of all readout parameters were blinded to the experimental groups. To reduce the variability of our outcome parameters caused by sex differences and to thereby decrease group sizes, we used only male mice in the study. In recent studies, severe effects of sex differences on infarct sizes and the inflammatory response were found [22]. Mice were excluded from endpoint analyses for the following pre-specified reasons: (1) death before the predefined experimental endpoint; (2) drop out score (weight loss, general condition, spontaneous behavior); (3) operation time > 10 min (to exclude the influence of prolonged anesthesia and increase group comparability). Numbers of included/omitted mice are shown in additional file 1. For induction of MCAO, mice were anesthetized with 2% isoflurane in O_2_ (v/v), and we injected 200 mg/kg of body weight Metamizole subcutaneous, and Lidocaine gel was used on the margin of the wound as analgesia. To maintain core body temperature close to 37 °C throughout surgery, a servo-controlled heating blanket was used. After a midline neck incision, a standardized silicon rubber-coated no. 6.0 nylon monofilament (6023910PK10; Doccol, Sharon, MA, USA) was inserted into the right common carotid artery and advanced via the internal carotid artery to occlude the origin of the MCA for 2, 3, or 4 h. For the 2 h MCAO/6 h reperfusion group, after 2 h, mice were re-anesthetized, and the occluding filament was removed to allow reperfusion. The operation time per animal did not exceed 10 min. Edema-corrected stroke volumes were assessed 2, 3, and 4 h after MCAO or after 2 h MCAO with 6 h reperfusion phase, based on 2,3,5-triphenyltetrazolium chloride (TTC)-, Nissl-, or Map2a/b staining (ImageJ software, National Institutes of Health). Sample size calculation was performed using estimates of the typical experimental brain infarct volume from previous studies, [23–25] a standard deviation of 20% to the respective mean values, a power of 90%, and a probability of a type I error of < 5%. Therefore, a group size of ≥ 9 was necessary to confidently see a difference of 30% in stroke size.

### Triphenyltetrazolium chloride (TTC) staining

Animals were sacrificed 2, 3, and 4 h after MCAO or after 2 h MCAO with 6 h reperfusion phase, and the brains were cut in three 2-mm-thick coronal sections. The slices were stained for 20 min at 37 °C with 2% TTC to visualize the infarctions. Edema-corrected infarct volumes were calculated by planimetry (ImageJ software, National Institutes of Health) [26].

### Animal treatment

Mice received 100 μg p0p/B antigen-binding fragment (Fab) i.v. immediately or 1 h after stroke induction to inhibit platelet GPIb. Controls received 100 μg rat IgG Fab [24]. For T-cell transfer experiments into *Rag1*^*−/−*^ mice, splenic T-cells were isolated from WT C57Bl/6 mice via MACS cell separation (CD4^+^ T-cell Isolation Kit, Miltenyi Biotec, Bergisch Gladbach, Germany). Cells were injected intravenously (750,000 cells/mouse) 1 day before MCAO.

### Histology, immunohistochemistry, and TUNEL assay

For histology, mice were anesthetized with isoflurane and sacrificed by decapitation. Brain tissue was cut in 2-mm-thick coronal sections, embedded in Tissue-Tek OCT compound and frozen. Brain sections were cut on a cryostat into 10-μm thin slices and used for all analysis. For immunohistochemistry, slices were post-fixated in 4% paraformaldehyde.

We stained mouse brains with Cresyl Violet (#C5042, Merck, Darmstadt, Germany) or antibodies against MAP2 (#ab32454, Abcam, Cambridge, UK, dilution 1:500), Ly-6B.2 (polymorphonuclear cells; MCA771GA, Bio-Rad, Hercules, CA, dilution 1:500), CD11b (macrophages/microglia; MCA711, Bio-Rad, Hercules, CA, dilution 1:100), CD4 (T-cells, #100506, BioLegend, San Diego, CA, dilution 1:50), CD8a (T-cells, #100724, BioLegend, San Diego, CA, dilution 1:50), NeuN (neurons; MAB377, Merck, Darmstadt, Germany, dilution 1:500), and TUNEL (In situ Cell Death Detection Kit, TMR red, 12156792910, Merck, Darmstadt, Germany) as described previously [24, 26, 27].

For all quantifications, identical brain sections at the level of the basal ganglia (0.5 mm anterior from bregma) were selected, and cell counting was performed from 3 subsequent slices or 1 slice (CD4^+^, CD8a^+^) of 4-5 different animals under a microscope (Leica DMi8 equipped with the DMC 2900/DFC 3000 G camera control and LAS X software (Leica, Wetzlar, Germany)). TUNEL-positive neurons were counted from 5 visual fields of identical cortical (regions 1–3) and subcortical (regions 4–5) regions at the level of the basal ganglia (0.5 mm anterior from bregma) from 5 subsequent brain slices of 4 different animals as described [28]. Negative controls for all histological experiments included omission of primary or secondary antibody and gave no signals (not shown).

### Statistical analyses

All data from animal experiments are given as box plots including median (Med) with the 25th percentile (25%), the 75th percentile (75%), minimum, and maximum. For statistical analysis, the GraphPad Prism 6 software package was used. Data were tested for Gaussian distribution with the D’Agostino-Pearson omnibus normality test and then analyzed by 1-way analysis of variance (ANOVA) with post hoc Bonferroni adjustment for *p* values or for nonparametric analysis compared by Kruskal–Wallis test with post hoc Dunn’s multiple comparisons test. If only 2 groups were compared, an unpaired, 2-tailed Student *t* test, or in the case of nonparametric distribution, the Wilcoxon–Mann–Whitney *U*-test, was applied. Probability values < 0.05 were considered to indicate statistically significant results.

## Results

### Targeting platelet GPIb reduces infarct progression under MCA occlusion

**Fig. 1:**
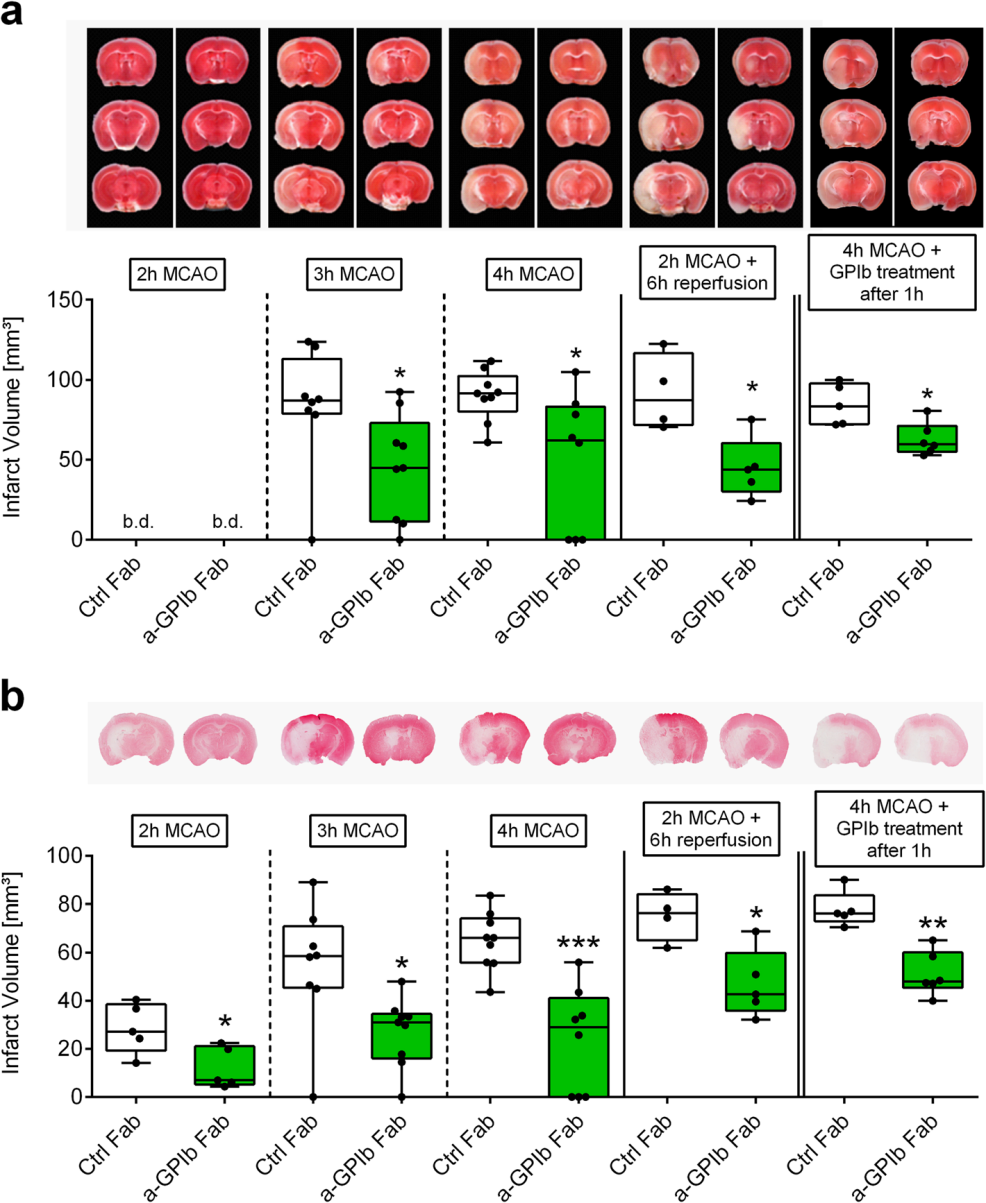
Blocking of GPIb delays ischemic brain damage. Representative images of coronal sections stained with **a** TTC, **b** Map2a/b 2, 3 and 4 h after MCAO or after 2 h of MCAO with additional 6 h of reperfusion in mice treated with rat IgG Fab (Ctrl Fab) or p0p/B Fab (a-GPIb Fab) immediately or 1 h after MCA occlusion. Infarcted areas are shown in white. Planimetric analyses were used to quantify the infarct volumes. Results are presented as box plots (*n* = 4–9). *P< 0.05, **P< 0.01, ***P< 0.001 between the indicated groups, 2-tailed Student *t* test or, in the case of nonparametric functional outcome, the Wilcoxon–Mann–Whitney *U*-test was applied. b.d., beyond detection level

**Fig. 2:**
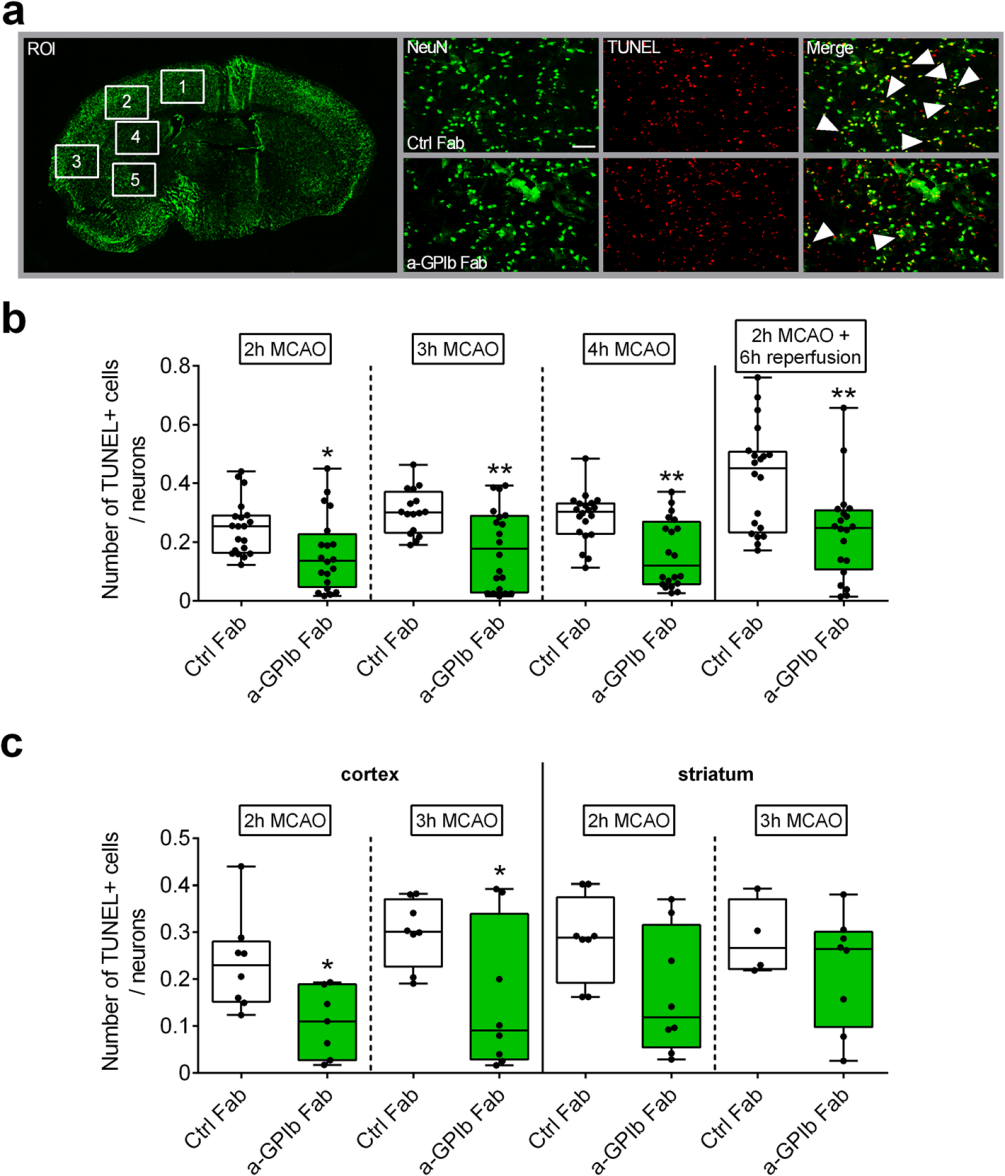
Neuronal apoptosis is reduced with GPIb treatment. **a** Schematic view of brain regions of interest (ROI) analyzed to quantify TUNEL^+^ cells/neurons and representative immunocytologic stainings. **b** Quantification of neurons stained with NeuN (Alexa488, green) and subjected to TUNEL assay (TMR red, red) in the ipsilateral hemisphere 2, 3, and 4 h after MCAO or after 2 h of MCAO with additional 6 h of reperfusion overall (ROI 1-5) and **c** divided in cortex (ROI 1 and 2: neocortex) and striatum (ROI 4 and 5: subcortex) stained with NeuN (Alexa488, green) and subjected to TUNEL assay (TMR red, red) in the ipsilateral hemisphere 2 and 3 h after MCAO in mice treated with rat IgG Fab (Ctrl Fab) or p0p/B Fab (a-GPIb Fab) using × 20 objective. Scale bar 100 μm (*n* = 16–20 slides). *P< 0.05, **P< 0.01 between the indicated groups, 2-tailed Student *t* test or, in the case of nonparametric functional outcome, the Wilcoxon–Mann–Whitney *U*-test was applied

We occluded the MCA by a filament in groups of animals for 2, 3, or 4 h and treated them immediately with anti-GPIb Fab fragments upon vessel occlusion. Strikingly, infarct volumes in mice treated with anti-GPIb-Fab were significantly reduced compared to control Fab-treated animals at the corresponding time points, as revealed by TTC (Fig. 1a), Nissl (Fig. S1), and MAP2 stainings (2 h MCAO: Ctrl Fab: Med. 27.2 (25%: 19.3; 75%: 38.5) mm^3^; anti-GPIb Fab: Med. 7.0 (25%: 5.2; 75%: 21.1) mm^3^, *P* < 0.05; 3 h MCAO: Ctrl Fab: Med. 58.5 (25%: 45.3; 75%: 70.9) mm^3^; anti-GPIb Fab: Med. 31.0 (25%: 16.1; 75%: 34.5) mm^3^, *P* < 0.05; 4 h MCAO: Ctrl Fab: Med. 66.1 (25%: 55.8; 75%: 74.2) mm^3^; anti-GPIb Fab: Med. 29.0 (25%: 0.0; 75%: 41.1) mm^3^, *P* < 0.001) (Fig. 1b). This means that GPIb blockade reduced infarct growth into the penumbra already under MCA occlusion. Furthermore, blocking of platelet GPIb led to a long-lasting reduction of infarct volumes that persisted into the reperfusion phase of 6 h (MAP2-staining: Ctrl Fab: Med. 76.3 (25%: 65.1; 75%: 84.1) mm^3^; anti-GPIb Fab: Med. 42.6 (25%: 35.9; 75%: 59.8) mm^3^, *P* < 0.05). Next, mice were treated therapeutically with a delay of 1 h after MCA occlusion. Again, a stroke-mitigating effect was seen at 4 h (MAP2-staining: Ctrl Fab: Med. 76.2 (25%: 73.0; 75%: 83.6) mm^3^; anti-GPIb Fab: Med. 47.9 (25%: 45.3; 75%: 60.1) mm^3^, *P* < 0.01). To prove that the apparent strong protective effect was specifically related to the cortical brain tissue at the border of the penumbra, which is supplied through residual collateral blood flow during MCA occlusion, we analyzed infarct areas divided into cortical and subcortical regions. In fact, in all analyzed groups, infarcted brain areas were especially reduced within the cortex closely matching the border of the penumbra, when anti-GPIb Fab was administered. In accordance with reduced infarct volumes, overall neuronal apoptosis (regions 1 to 5) was diminished within the ischemic hemispheres in the anti-GPIb-treated groups (Fig. 2a, b). In consistence with the structural and functional anatomy of collateral flow under the condition of residual perfusion during occlusion, fewer apoptotic neurons were found especially within the neocortex (regions 1 and 2) (2 h MCAO: Ctrl Fab: Med. 0.23 (25%: 0.15; 75%: 0.28); anti-GPIb Fab: Med. 0.11 (25%: 0.03; 75%: 0.19), *P* < 0.05; 3 h MCAO: Ctrl Fab: Med. 0.30 (25%: 0.23; 75%: 0.37); anti-GPIb Fab: Med. 0.09 (25%: 0.03; 75%: 0.34), *P* < 0.05), while the reduction was not significant in the subcortical zone (regions 4 and 5) (Fig. 2c).

### Recruitment and functional impact of T-cells to infarct progression

**Fig. 3:**
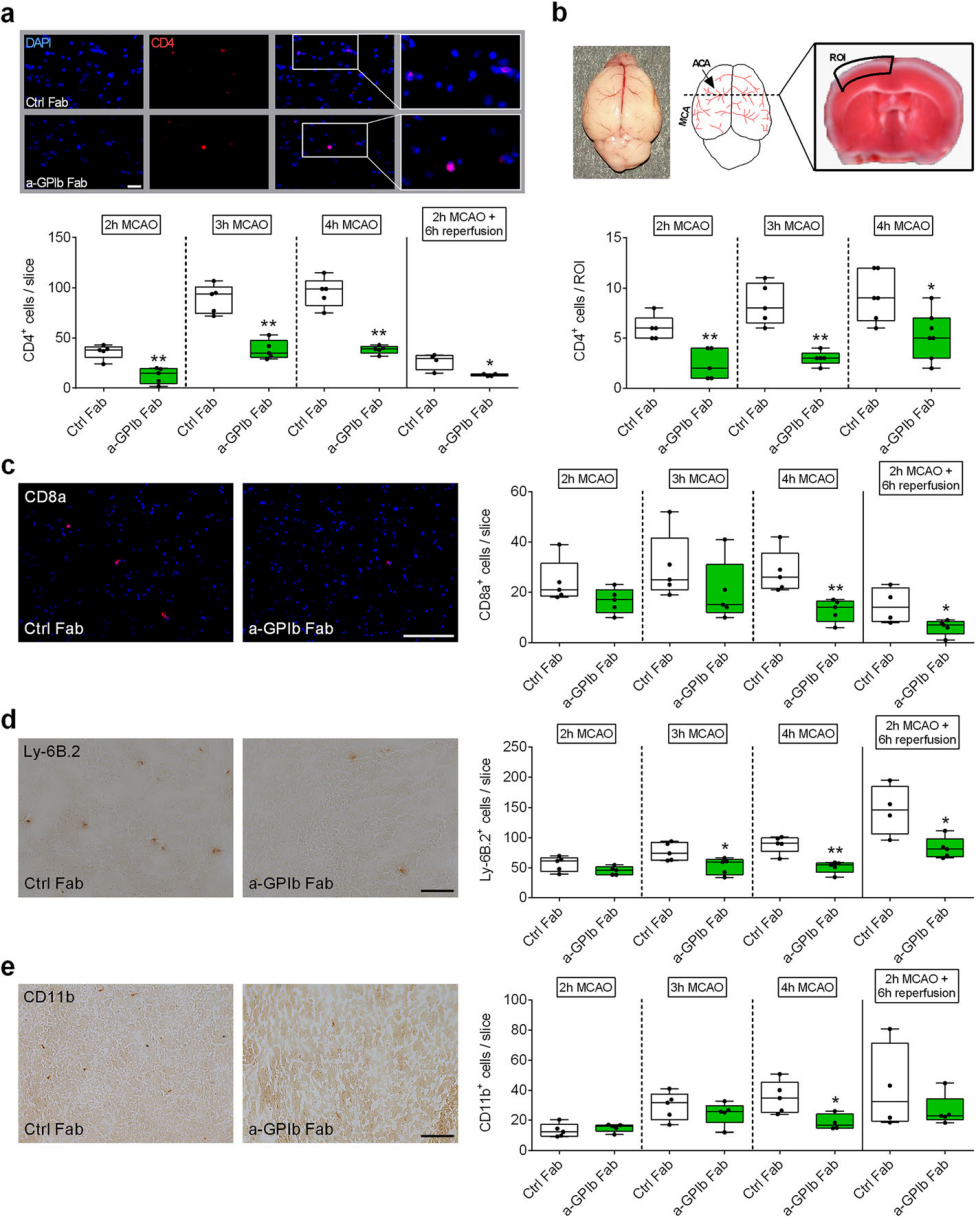
Blocking of platelet GPIb diminished ultra-early T-cell recruitment in the ischemic brain. **a** Representative immunocytologic stainings (top) and quantification (bottom) of brain-infiltrating CD4-positive T lymphocytes (Cy3, red), nuclei (DAPI, blue) in the whole ipsilateral hemisphere 2, 3, and 4 h after MCAO or after 2 h of MCAO with additional 6 h of reperfusion in mice treated with rat IgG Fab (Ctrl Fab) or p0p/B Fab (a-GPIb Fab) using × 20 objective. Scale bar 100 μm (*n* = 4–5). **b** (top) Native whole mouse brain with schematic overview of ACA and MCA territories. The ROI used for T-cell quantification is shown on a TTC-stained coronal brain section (2 h MCAO). (bottom) Quantification of brain-infiltrating CD4-positive T lymphocytes (Cy3, red), nuclei (DAPI, blue) in the ROI at 2, 3, and 4 h after MCAO in mice treated with rat IgG Fab (Ctrl Fab) or p0p/B Fab (a-GPIb Fab) using × 20 objective (*n* = 5). Representative images (left) and quantification (right) of **c** CD8a-positive cells (CD8a (Alexa 647, red), nuclei (DAPI, blue), **d** Ly-6B.2-positive cells, or **e** CD11b-positive cells (3,3-Diaminobenzidin (DAB)) in the ipsilateral hemisphere 2, 3, and 4 h after MCAO or after 2 h of MCAO with additional 6 h of reperfusion in mice treated with rat IgG Fab (Ctrl Fab) or p0p/B Fab (a-GPIb Fab) using × 20 objective. Scale bar 100 μm (*n* = 4–5). *P< 0.05, **P< 0.01 between the indicated groups, 2-tailed Student *t* test or, in the case of nonparametric functional outcome, the Wilcoxon–Mann–Whitney *U*-test was applied. ACA, anterior cerebral artery; MCA, middle cerebral artery; ROI, region of interest

Next, we assessed the effect of GPIb inhibition on the accumulation of immune cells during the occlusion condition in the brain. At the earliest examination time, i.e., after 2 h MCA occlusion, anti-GPIb treated mice showed reduced numbers of CD4^+^ T lymphocytes (Ctrl Fab: Med. 38.0 (25%: 30.5; 75%: 41.0); anti-GPIb Fab: Med. 15.0 (25%: 4.5; 75%: 19.5), *P* < 0.01) but not of CD8a, Ly-6B.2, or CD11b positive cells compared to control mice (Fig. 3a, c, d, e). In line with reduced cortical volume of infarction and improved neuronal survival in the neocortex, CD4^+^ T-cell counts were significantly reduced within this region in anti-GPIb-treated mice (2 h MCAO: Ctrl Fab: Med. 6.0 (25%: 5.0; 75%: 7.0); anti-GPIb Fab: Med. 2.0 (25%: 1.0; 75%: 4.0), *P* < 0.01; 3 h MCAO: Ctrl Fab: Med. 8.0 (25%: 6.5; 75%: 10.5); anti-GPIb Fab: Med. 3.0 (25%: 2.5; 75%: 3.5), *P* < 0.01; 4 h MCAO: Ctrl Fab: Med. 9.0 (25%: 6.8; 75%: 12.0); anti-GPIb Fab: Med. 5.0 (25%: 3.0; 75%: 7.0), *P* < 0.05) (Fig. 3b). With prolonged vessel occlusion, the numbers of T lymphocytes (CD4^+^ and CD8a^+^), Ly-6B.2^+^, and CD11b^+^ cells were significantly reduced in the anti-GPIb-treated group (Fig. 3a, c, d, e). No B cell recruitment at all was measurable during MCA occlusion (not shown). These data demonstrate that GPIb-blockade affected early CD4^+^ T lymphocyte recruitment into the ischemic brain, importantly, already during the hyperacute stage under the condition of occlusion.

**Fig. 4:**
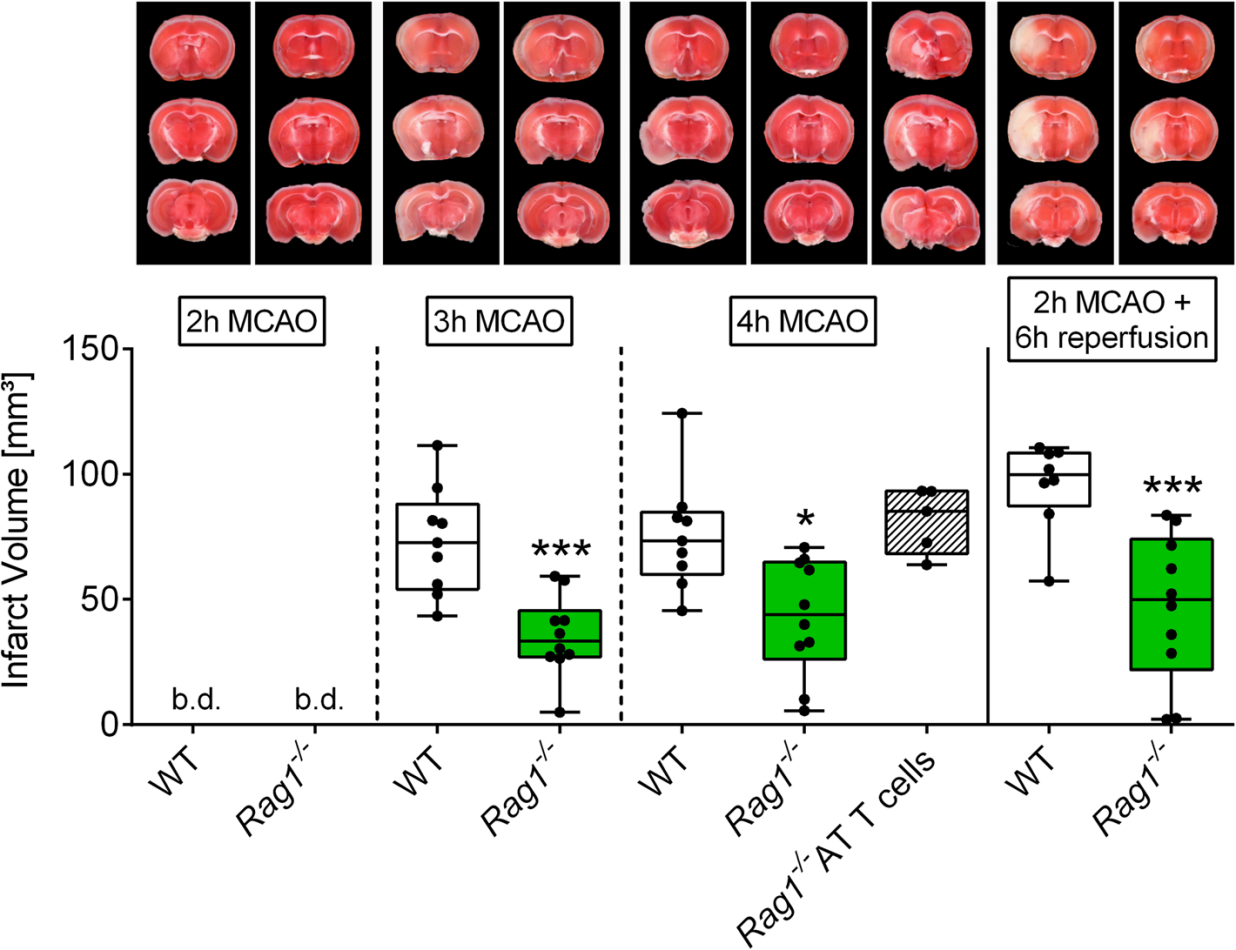
T-cells are required to promote infarct growth during MCAO. Representative images of coronal sections stained with TTC 2, 3, and 4 h after MCAO or after 2 h of MCAO with additional 6 h of reperfusion in *Rag1*^*−/−*^ or WT mice and representative images of coronal sections stained with TTC 4 h after MCAO in *Rag1*^*−/−*^ mice with adoptively transferred T-cells (AT T-cells). Infarcted areas are shown in white. Planimetric analysis was used to quantify the infarct volume. Results are presented as box plots (*n* = 5–10). *P< 0.05, and ***P< 0.001 between the indicated groups, 2-tailed Student *t* test or, in the case of nonparametric functional outcome, the Wilcoxon–Mann–Whitney *U*-test was applied. Nonparametric functional outcome between 3 groups were compared by Kruskal–Wallis test with post hoc Dunn’s multiple comparisons test. b.d., beyond detection level

To unravel the contribution of CD4^+^ T lymphocytes to infarct progression under occlusion, we subjected *Rag1*^*−/−*^ mice, which lack B and T lymphocytes, to extended occlusion times. Similar to our findings after GPIb blockade, in *Rag1*^*−/−*^ mice, infarct progression was significantly mitigated (3 h MCAO: WT: Med. 72.7 (25%: 54.0; 75%: 88.1) mm^3^; *Rag1*^*−/−*^: Med. 33.4 (25%: 27.0; 75%: 45.5) mm^3^, *P* < 0.001; 4 h MCAO: WT: Med. 73.4 (25%: 60.0; 75%: 84.8) mm^3^; *Rag1*^*−/−*^: Med. 44.0 (25%: 26.1; 75%: 65.0) mm^3^, *P* < 0.01) indicating salvage of penumbral brain tissue under occlusion (Fig. 4). Of note, adoptive CD4^+^ T-cell transfer into *Rag1*^*−/−*^ mice prior to MCA occlusion increased infarct volumes to the levels seen in wildtype controls at 4 h of MCAO (WT: Med. 73.4 (25%: 60.0; 75%: 84.8) mm^3^; *Rag1*^*−/−*^ AT T-cells: Med. 85.3 (25%: 68.3; 75%: 93.3) mm^3^, *P* > 0.05), thereby confirming a key role of CD4^+^ T-cells in the process of infarct growth into the penumbra under occlusion, i.e., ultra-early stroke.

## Discussion

As principal finding, we show that it is possible to retard infarct progression into the penumbra already during the ultra-early phase under MCA occlusion by either blocking platelet GPIb or by lymphocyte deficiency in mice. So far, the strong association between declining collateral perfusion and progressive loss of the penumbra has directed attention towards therapeutic measures which tackle collateral flow [9, 10, 29]. These reach from supportive medical care, such as adequate hydration and avoidance of fluctuations in blood pressure, induced hypertension, application of perfluorocarbons, transient descending aortic balloon occlusion to sensory/sphenopalatine ganglion stimulation, among others [9, 10, 29]. The basic idea was to “freeze” the penumbra before recanalization by *augmenting* collateral flow through these physical and pharmacological measures [10].

Our present study paves the way for an alternative treatment approach by targeting thrombo-inflammation to preserve the penumbra already under MCA occlusion. As C57Bl/6 mice exhibit robust collaterals between the anterior-middle-posterior cerebral artery territories as in humans, [9] platelets and therapeutic anti-GPIb Fab fragments have access to the ischemic penumbra by retrograde collateral blood flow. GPIb facilitates tethering of platelets to the vessel wall and their interaction with immune cells, whereas further activation steps, mediated by other receptors are necessary to induce thrombus formation. Therefore, we hypothesize that the protective effects are not primarily due to vascular hemodynamic factors increasing the capacity of the macrovascular collateral anastomoses upstream, but rather by blocking thrombo-inflammation within the downstream microvasculature. Thrombo-inflammation has been identified as a major component of ischemia/reperfusion (I/R) injury in cerebral ischemia [19] in which platelet and T-cell interactions cause brain tissue damage [2, 18, 30]. For the first time, we show here that a similar process is set into motion already during the phase of macrovascular occlusion, and thus amenable to treatment prior to recanalization. Our notion is supported by the fact that *Rag1*^*−/−*^ mice were likewise protected from penumbral tissue loss, i.e., infarct progression under MCA occlusion. Recently, the deleterious contribution of lymphocytes to acute human stroke was confirmed in studies in patients who received fingolimod, which induces rapid lymphopenia, in conjunction with thrombolysis or MTE, and showed a mitigated secondary infarct growth [31, 32]. Moreover, we sampled ischemic blood from the pial collateral circulation within the center of the penumbra and during occlusion in hyperacute human stroke. We found locally increased leukocyte counts in the post-occlusive arterial blood indicating directly that a local inflammatory response occurs that involves lymphocytes [17].

Novel treatments in acute stroke patients are eagerly awaited bridging the gap between stroke onset and arrival at secondary/tertiary stroke centers with the ability for MTE in patients with ICA/MCA in whom thrombolysis is inefficient and infarcts progress into the penumbra upon transportation [6, 7, 33, 34]. Our experimental study opens a potential new therapeutic avenue by showing that platelet GPIb and T-cells are involved in penumbral tissue loss before recanalization, similar to ischemia/reperfusion injury after recanalization as shown before. Interfering with thrombo-inflammation to delay organ infarction under fully occlusive ischemia is a promising novel approach which may be relevant not only to ischemic stroke but particularly also to myocardial infarction.

### Study limitations

Our study is a proof of principle study in young male mice; therefore, to confirm the potential of our promising result for clinical translation, a number of issues need to be clarified in further studies: (i) gender can have a significant impact on stroke outcome in rodents as well as in humans [35], consequently experiments need to be repeated in female mice; (ii) considering that the typical stroke patient is elderly with comorbidity, similar to the sequential previous approach on the role of GPIb in ischemia/reperfusion injury experiments in old and comorbid mice to confirm the therapeutic effect is required [18, 36]; (iii) defining platelet-dependent safeguard mechanisms including the necessity of GPIb to maintain hemostasis after prolonged cerebral ischemia. In previous studies addressing the role of platelets in cerebral I/R injury, blocking of their GPIb and GPVI receptors was safe also in long-term experiments while blocking of GPIIb/IIIa-mediated platelet aggregation or lack of platelet granules led to severe intracranial hemorrhages during reperfusion [18, 25, 37].

To unravel the contribution of T lymphocytes to infarct growth under occlusion, we subjected *Rag1*^*−/−*^ mice to extended occlusion times and performed adoptive transfer experiments of CD4^+^ T-cells. Consequently, as done before in the context of I/R injury, further studies are needed in order to unravel the contribution of additional T-cell populations [19].

## Conclusions

This study demonstrated that progressive brain infarction can be delayed by blocking detrimental lymphocyte/platelet responses already during occlusion paving the way for ultra-early treatment strategies in hyper-acute stroke before recanalization.

## Supplementary Information

**Additional file 1: Table S1**. Experimental Groups. **Supplemental Figure S1.**

## Data Availability

The datasets used and/or analyzed during the current study are available from the corresponding author on reasonable request.

## Abbreviations

MTE: Mechanical thrombectomy
I/R: Ischemia/reperfusion
MCAO: Occlusion of the middle cerebral artery
GPIb: Glycoprotein Ib
