# Supplementary material for: Platelets and lymphocytes drive progressive penumbral tissue loss during middle cerebral artery occlusion in mice
Source: J Neuroinflammation. 2021 Feb 18;18:46. doi: 10.1186/s12974-021-02095-1 (PMC7890632; doi:10.1186/s12974-021-02095-1)
Supplement: Supplementary file 1 — Additional file 1: Table S1. Experimental Groups. Supplemental Figure S1. [file 12974_2021_2095_MOESM1_ESM.docx]

**Additional file 1**

**Platelets and lymphocytes drive progressive penumbral tissue loss during middle cerebral artery occlusion in mice**

Michael K. Schuhmann^1^*, Michael Bieber^1^*, Maximilian Franke^1^, Alexander M. Kollikowski^2^, David Stegner^3,4^, Katrin G. Heinze^3^, Bernhard Nieswandt^3,4^, Mirko Pham^2^, Guido Stoll^1^

*contributed equally to this study

^1^Department of Neurology, University Hospital Würzburg, Würzburg, Germany; ^2^Department of Neuroradiology, University Hospital Würzburg, Würzburg, Germany; ^3^Rudolf Virchow Center for Integrative and Translational Bioimaging, University of Würzburg, Würzburg, Germany; ^4^Institute of Experimental Biomedicine, University Hospital Würzburg, Würzburg, Germany.

**Correspondence to: Guido Stoll, E-mail:** stoll_g@ukw.de

**Table S1** Experimental Groups

| **Time period** | **C57Bl/6 N**  **IgG Fab (Ctrl Fab) treatment**  **Incl. / omitted*** | **C57Bl/6 N**  **anti-GPIb Fab (a-GPIb Fab) treatment**  **Incl. / omitted*** | **C57Bl/6 J**  **WT**  **Incl. / omitted*** | ***Rag1^-/-^***  **Incl. / omitted*** |
| --- | --- | --- | --- | --- |
| **Infarct size (TTC, Nissl, MAP2a/b)** | | | | |
| 2h MCAO | 5 / 0 | 5 / 0 | 8 / 0 | 9 / 0 |
| 3h MCAO | 8 / 2 | 9 / 1 | 9 / 0 | 10 / 0 |
| 4h MCAO | 9 / 1 | 8 / 2 | 9 / 0 | 10 / 0 |
| 2h MCAO + 6h reperfusion | 4 / 1 | 5 / 0 | 8 / 2 | 10 / 0 |
| 4h MCAO + GPIb treatment after 1h | 5 / 0 | 6 / 0 |  |  |
| adoptive transfer of CD4^+^ T cells |  |  |  | 5 / 0 |

*all animals omitted from an endpoint analysis were excluded due to death after treatment or due to bad clinical condition. No mice had to be excluded due to unsatisfactory surgery.

Supplemental Figure


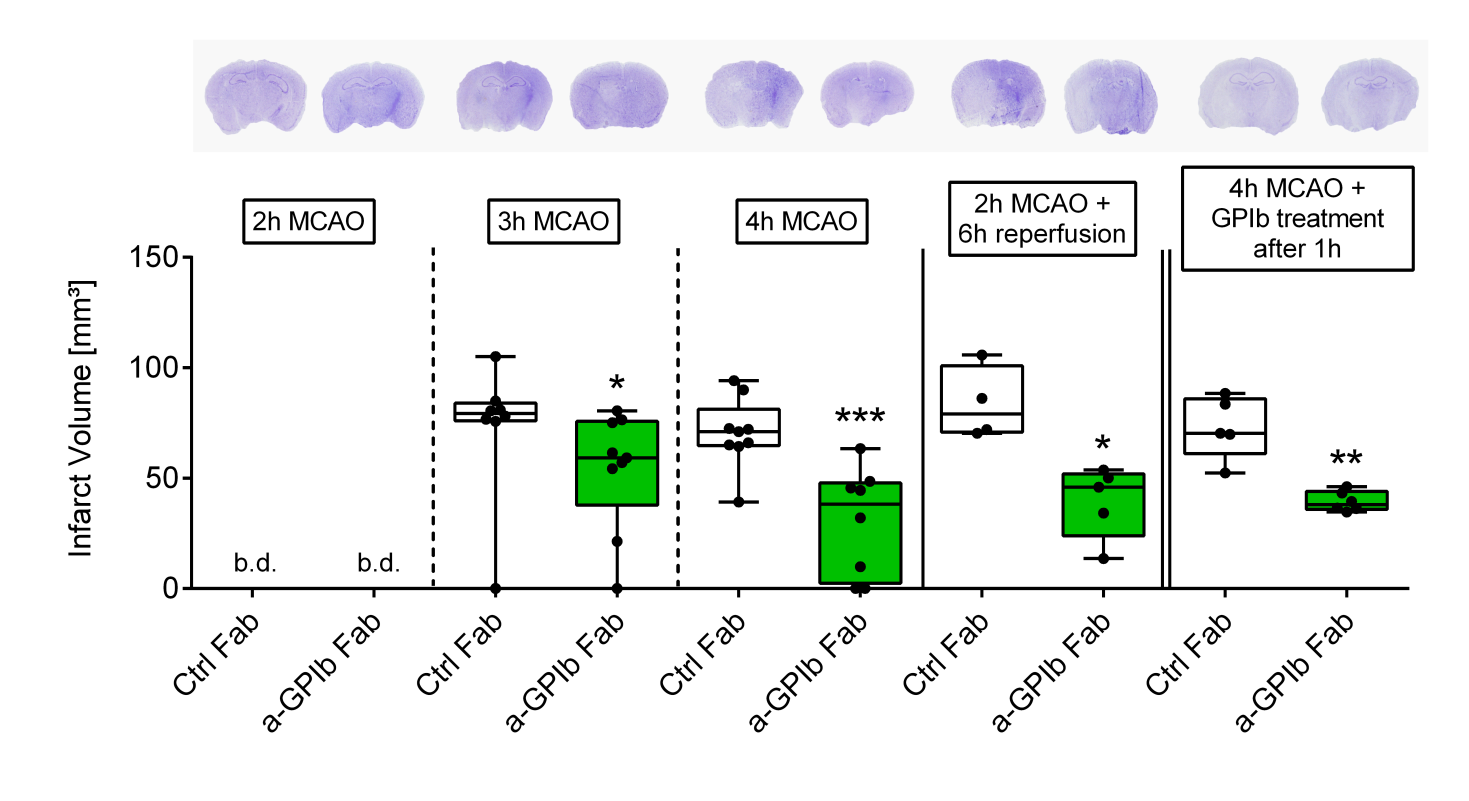
**Fig. S1** Blocking of GPIb delays ischemic brain damage. Representative images of coronal sections stained with Nissl 2, 3 and 4 h after MCAO or after 2 h of MCAO with additional 6 h of reperfusion in mice treated with rat IgG Fab (Ctrl Fab) or p0p/B Fab (a-GPIb Fab) immediately or 1 hour after MCA occlusion. Infarcted areas are shown in white. Planimetric analyses were used to quantify the infarct volumes. Results are presented as box plots (n=4-9). **P*< 0.05, ***P*< 0.01, ****P*< 0.001 between the indicated groups, 2-tailed Student *t* test or in the case of nonparametric functional outcome, the Wilcoxon–Mann–Whitney *U*-test was applied. b.d., beyond detection level
